# Non-endothelial endothelin counteracts hypoxic vasodilation in porcine large coronary arteries

**DOI:** 10.1186/1472-6793-11-8

**Published:** 2011-05-15

**Authors:** Elise R Hedegaard, Edgaras Stankevicius, Ulf Simonsen, Ole Fröbert

**Affiliations:** 1 Department of Pharmacology, Aarhus University, Wilhem Meyers Allé 4, Bldg. 1240, 8000 Aarhus C, Denmark; 2 Department of Physiology, Medical Academy, Lithuanian University of Health Sciences, A. Mickevičiaus 9, 44307 Kaunas, Lithuania; 3 Department of Cardiology, Örebro University Hospital, Södra Grev Rosengatan, 701 85 Örebro, Sweden

## Abstract

**Background:**

The systemic vascular response to hypoxia is vasodilation. However, reports suggest that the potent vasoconstrictor endothelin-1 (ET-1) is released from the vasculature during hypoxia. ET-1 is reported to augment superoxide anion generation and may counteract nitric oxide (NO) vasodilation. Moreover, ET-1 was proposed to contribute to increased vascular resistance in heart failure by increasing the production of asymmetric dimethylarginine (ADMA). We investigated the role of ET-1, the NO pathway, the potassium channels and radical oxygen species in hypoxia-induced vasodilation of large coronary arteries.

**Results:**

In prostaglandin F_2α _(PGF_2α_, 10 μM)-contracted segments with endothelium, gradual lowering of oxygen tension from 95 to 1% O_2 _resulted in vasodilation. The vasodilation to O_2 _lowering was rightward shifted in segments without endothelium at all O_2 _concentrations except at 1% O_2_. The endothelin receptor antagonist SB217242 (10 μM) markedly increased hypoxic dilation despite the free tissue ET-1 concentration in the arterial wall was unchanged in 1% O_2 _versus 95% O_2. _Exogenous ET-1 reversed hypoxic dilation in segments with and without endothelium, and the hypoxic arteries showed an increased sensitivity towards ET-1 compared to the normoxic controls. Without affecting basal NO, hypoxia increased NO concentration in PGF_2α_-contracted arteries, and an NO synthase inhibitor, L-NOARG,(300 μM, N^G^-nitro-L-Arginine) reduced hypoxic vasodilation. NO-induced vasodilation was reduced in endothelin-contracted preparations. Arterial wall ADMA concentrations were unchanged by hypoxia. Blocking of potassium channels with TEA (tetraethylammounium chloride)(10 μM) inhibited vasodilation to O_2 _lowering as well as to NO. The superoxide scavenger tiron (10 μM) and the putative NADPH oxidase inhibitor apocynin (10 μM) leftward shifted concentration-response curves for O_2 _lowering without changing vasodilation to 1% O_2_. PEG (polyethylene glycol) catalase (300 u/ml) inhibited H_2_O_2 _vasodilation, but failed to affect vasodilation to O_2 _lowering. Neither did PEG-SOD (polyethylene glycol superoxide dismutase)(70 u/ml) affect vasodilation to O_2 _lowering. The mitochondrial inhibitors rotenone (1 μM) and antimycin A (1 μM) both inhibited hypoxic vasodilatation.

**Conclusion:**

The present results in porcine coronary arteries suggest NO contributes to hypoxic vasodilation, probably through K channel opening, which is reversed by addition of ET-1 and enhanced by endothelin receptor antagonism. These latter findings suggest that endothelin receptor activation counteracts hypoxic vasodilation.

## Background

The systemic vascular response to hypoxia is thought to be vasodilation [1,2], although lowering oxygen (O_2_) from 95% to 1-5% O_2 _either induced or enhanced constriction in canine [3,4] and sheep [1,5] large coronary arteries, while moderate hypoxia (12-40%) O_2 _was reported to induce transient contractions in human and porcine coronary arteries, and only vasodilation in response to anoxia [1,6]. Reports also indicate that the potent vasoconstrictor endothelin-1 (ET-1) is released from the vasculature during hypoxia [7,8]. ET-1 is critical in the development of cardiovascular diseases such as pulmonary hypertension, atherosclerosis, hypertension, and heart failure where hypoxia is a central feature [9]. ET-1 was reported to augment superoxide anion generation in human endothelial cells, suggesting a mechanism for enhanced susceptibility to atherosclerosis [10], and it was found that asymmetric dimethylarginine (ADMA) and ET-1 levels correlate with the extent of intimal hyperplasia [11]. Moreover, ET-1 was proposed to contribute to increased vascular resistance in heart failure by increasing the production of ADMA [12]. We found that the plasma concentration of ADMA rises following coronary angioplasty in patients with myocardial infarction and in patients with stable angina pectoris which are events associated with localised and general tissue hypoxia [13]. ET-1 may also counteract nitric oxide (NO) vasodilation by increasing the levels of free radical oxygen species [10,14,15]. Thus, superoxide anions may react with NO to generate peroxynitrite (ONOO^-^), and hence lower the NO concentration, or be converted by superoxide dismutase to hydrogen peroxide [16]. Thus, many mechanisms have been suggested to contribute to the hypoxic response in coronary arteries. Therefore, we have revisited the role of endothelial factors in the arterial response to hypoxia.

In the present study, we hypothesized that endothelium-derived factors modulate hypoxic vasodilation in large porcine coronary arteries. To address this hypothesis the following measurements were performed: (1) the role of endothelin was investigated by functional studies in isolated coronary arteries and measurement of ET-1 in the vascular wall, (2) the role of NO was evaluated by use of NO synthase and guanylyl cyclase inhibitors, and simultaneous measurements of the NO concentration and vascular contractility were performed, (3) ADMA levels in the vascular interstitial fluid were measured, (4) the involvement of the endothelial cell layer in acute hypoxic vasodilation was investigated by oxygen lowering performed in coronary arteries with and without endothelium, (5) involvement of potassium channels was investigated by adding a potassium channel blocker, tetraethylammonium (TEA), (6) a role for radical oxygen species was investigated by addressing the effect of scavengers of superoxide and H_2_O_2_, as well as inhibitors of the mitochondrial electron transport.

The precise oxygen tension in a large coronary artery wall is relatively unknown. In large arterial preparations e.g. rabbit and porcine aorta exposed to 21% O_2_, O_2 _tension was found to fall and reach very low levels in media of the vascular wall [17]. Therefore, in the present study we have chosen to gradually lower organ bath oxygen tension from 95% O_2 _(722 mm Hg) to 1% O_2 _(7.6 mm Hg).

## Methods

### Ethics Statement

Hearts from Landrace-Yorkshire hogs were obtained at a local slaughterhouse. All experiments conformed to the European Convention for the Protection of Vertebrate Animals used for Experimental and other Scientific Purposes [18].

### Tissue

Immediately after sacrifice the aorta was cannulated and the coronary circulation perfused with physiological saline solution (PSS) containing glucose 5.5 mM, bubbled with 5% CO_2 _in O_2 _and buffered with HEPES (for composition see [19]). The hearts were bathed in PSS at 5°C for approximately 2 hours until the start of the experiment. The left anterior descending coronary artery (LAD) was carefully dissected and the proximal 3-4 cm of the artery was left intact (pressure myograph) or cut into a maximum of 4 segments each being 2 mm long, and each of the segments was used in different treatments. Therefore, each experiment equals one pig (wire myograph). In some experiments the endothelium was removed by gentle rubbing of the lumen with a thin wooden stick.

### Functional myograph studies

**Figure 1:**
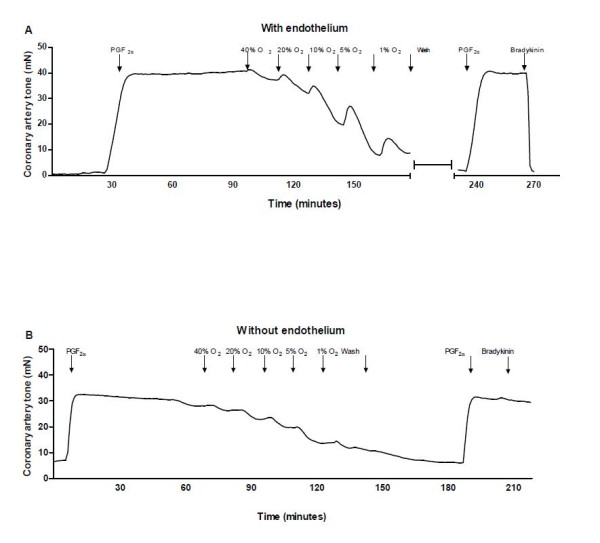
**Original trace illustrating the relaxation induced by lowering the oxygen concentration**. (A) Pig coronary artery with endothelium, and (B) without endothelium. The arteries were contracted with PGF_2α _(10 μM), and O_2 _was gradually lowered, then the bath solution was exchanged (wash), the artery was reoxygenated and after a new contraction with PGF_2α _endothelial function was evaluated by adding bradykinin (30 nM).

Coronary artery segments were mounted for isometric tension recordings in a 4-chamber wire myograph (DMT, Tissue Bath System 700MO, Aarhus, Denmark) containing 5°C PSS bubbled with 95% O_2_, 5% CO_2_. Temperature was raised to 37°C and the arteries were normalized according to a standard procedure [20]. Arteries were allowed to equilibrate for approximately 30 minutes. Before running the experiment, preparation viability was examined by exposing arteries to potassium-rich PSS (K^+^PSS, 123.5 mM). The dilatory response to hypoxia was evaluated following a stable contractile response to prostaglandin F_2α _(PGF_2α_) 10 μM. After the experiment arteries were contracted with PGF_2α _(10 μM) and removal of the endothelial layer was evaluated by lack of response (<10% vasodilation) to bradykinin 30 nM (see Figure 1). Appropriate gas composition was obtained by mixing 95% O_2_, 5% CO_2 _with 95% N_2_, 5% CO_2 _using a gasmixer (Digamix, H. Wösthoff GmbH, Bochum, Germany). The organ bath was bubbled through glass tubes with pimpstone allowing rapid equilibration of the oxygen tension set by the pump. The gas tension was set to 95% O_2 _(722 mm Hg), 40% O_2 _(304 mm Hg), 20% O_2 _(152 mm Hg), 10% O_2 _(76 mm Hg), 5% O_2 _(38 mm Hg), and 1% O_2 _(7,6 mm Hg). Oxygen concentration was measured with an oxygen electrode (Unisense, Aarhus, Denmark), and the measured oxygen concentrations were 92.4 ± 0.7%, 39.6 ± 0.4%, 20.2 ± 0.2%, 11.0 ± 0.2%, 5.8 ± 0.2%, and 1.2 ± 0.3% (n = 8).

The oxygen curve was performed by maintaining the oxygen level at a fixed concentration until steady state before changing to the next level. We investigated the role of the endothelin receptor antagonist SB217242 (10 μM), the competitive NO synthase inhibitor N^G^-nitro-L-Arginine (L-NOARG, 300 μM), the potassium-selective ion channel blocker TEA (10 μM), the free radical scavenger tiron (10 μM), the putative NADPH oxidase inhibitor apocynin (10 μM), the catalyst for H_2_O_2 _decomposition PEG catalase (300 u/ml), the catalyst for dismutation of superoxide PEG-SOD (70 u/ml), and the mitochondrial inhibitors rotenone (1 μM), and antimycin A (1 μM). These were incubated 30 minutes prior to PGF_2α _contraction. We performed concentration-response curves for ET-1 (10^-11 ^- 3 × 10^-8 ^M), and the influence of oxygen and NO on the arteries was examined. In another set of experiments, the arteries were normalized and tested for the presence of endothelium before an inhibitor of guanylyl cyclase 1H-[1,2,4]oxadiazolo[4,3-a]quinoxalin-1-one (ODQ) (3 μM) was added and left until steady state before arteries were contracted with either PGF_2α _(1 μM) or ET-1 in appropriate amounts (1-10 nM) to reach similar levels of contraction. Oxygen or NO concentration-response curves were performed.

Tissue concentrations of ET-1 were determined in arterial segments mounted in wire myographs and contracted with 10 μM PGF_2α_, and exposed to hypoxia or oxygenation for 60 min. After treatment the arteries were frozen in cold acetone and dry ice at -78°C. Before homogenization the tissue was allowed to equilibrate at room temperature for 2 hours before the dried tissue was weighed. The samples were homogenized in EIA buffer from the ET-1 ELISA kit using a Precellys 24 homogenizer (Bertin Technologies, Montigny-le-Bretonneux, France). The samples were exposed to 3 cycles at 5000 rpm for 30 seconds each. After homogenization the samples were centrifuged at 10.000 rpm for 10 min at 4°C, and the supernatant was collected and kept at -80°C. The ET-1 content in each sample was quantified with an ELISA assay (Immuno-Biological Laboratories Co., Ltd, Japan) according to the manufacturer's instructions. This assay is a solid phase sandwich ELISA using 2 kinds of highly specific antibodies. Tetra Methyl Benzidine (TMB) was used as colouring agent (chromogen); the strength of colouring was proportional to the quantity of ET-1.

### Simultaneous measurements of NO concentration and force

For simultaneous measurement of force and NO concentration, an NO sensitive microelectrode (ISONOP30, World Precision Instruments, Stevenage, UK) was used. The calibration of the electrode was performed at 21% and 1% O_2 _by use of an NO solution and then introduced into the lumen of the artery mounted in a single channel wire myograph as previously described [19,21]. To test selectivity of the electrodes, a lack of response to sodium nitrite of up to 10 μM was regarded as evidence for an intact coating of the electrode. Noradrenaline is oxidized on carbon fibres if electrode coating is damaged, and electrodes were discarded if noradrenaline (0.5 μM), in the absence of vascular tissues, increased electrode current.

Coronary arterial segments 2 mm in length were suspended in a wire myograph (DMT, Tissue Bath System) for force measurements and the microelectrode was introduced into the lumen. To investigate the role of basally-released NO, simultaneous measurements of force and NO concentration were performed in both the absence and presence of a scavenger of free NO, oxyhaemoglobin (10 μM). To evaluate the effect of agonist induced relaxation and NO release, artery rings were contracted with PGF_2α_, 10 μM, and bradykinin was added.

### Functional testing and interstitial concentration of ADMA

Two cm long cylindrical arterial segments were mounted at both ends on a stainless steel cannulae and fastened with sutures in PSS bubbled with 5% CO_2 _in O_2 _in a pressure myograph (110P XL, DMT). The temperature was raised to 37C. Previous to the measurements the segments were stretched to the *in situ *length using a micrometer device. A transmural pressure of 40 mm Hg was applied for a 1-hour stabilizing period as well as during the experiments. We previously found this to be optimal in terms of coronary arterial response to an agonist in a pressure myograph [22]. The external diameter of the arterial segment was automatically determined by video imaging at a frequency of 20 Hz. The internal pressure was controlled by adjustment of two reservoirs containing PSS mounted on a pressure column and connected to the cannulae. Pressure transducers close to the "arterial" end of each cannula measured the internal pressure. Transmural pressure, the outer diameter, and a video image of the arterial segment were continuously sampled and stored on computer (Myodaq software, version 2.03, DMT). Before and after experiments the video dimension analyser was calibrated by use of a 3000 × 3000 μm^2 ^phantom in the horizontal and vertical directions.

After a stabilising period in the organ bath, artery tone was induced with PGF_2α_. When a stable constriction was established, hypoxia was induced by adjusting the gas concentrations from 5% CO_2 _in O_2 _(organ bath PO_2_>650 mm Hg, ISO2-D, WPI, Fl) to 5% CO_2 _in N_2 _resulting in 1% O_2 _in the organbath (PO_2 _30 mm Hg). The influence of 60 min of hypoxia was studied. Oxygenated conditions were re-established by switching back to PSS equilibrated with 5% CO_2 _in O_2 _and the arterial response during the following 60 min was recorded (reoxygenation period). Organ bath pH was constant at 7.4.

In other experiments a microdialysis catheter (CMA/7, CMA, Sweden) was placed in the smooth muscle interstitium of a coronary artery mounted in the pressure myograph as previously described [23]. The microdialysis catheter has a 6 kDa molecular cut-off and an outer diameter of 0.24 mm. Perfusion of the catheter was started immediately after mounting at a rate of 0.3 μl/min with isotonic saline. Thirty-minute samples of dialysate (9 μl/sample) were collected. There was a delay of 12 min between the passage of the perfusate through the microdialysis catheter and collection in vials. This time delay was considered in the calculations and all data are presented in real time. Microdialysis samples were collected 60 min prior to hypoxia, during hypoxia, and during reoxygenation. In a subset of experiments ADMA 10 μM was added to the organ bath at baseline.

Concentrations of dialysate ADMA concentrations were determined by high-performance liquid chromatography (HPLC, fluorescence detector) and precolumn derivation with o-pthaldialdehyde according to previous studies [24,25]. Standard curves for ADMA were constructed before each analysis.

### Statistics

Values are presented as mean ± SEM and number of vessels (one per pig). Because the coronary artery diameter varied between pigs, the steady state diameters induced by PGF_2α _were used as an internal standard (100%). Two-way analyses of variance and paired t-tests were used to test for differences between groups. Differences were considered statistically significant when P < 0.05.

## Results

**Figure 2:**
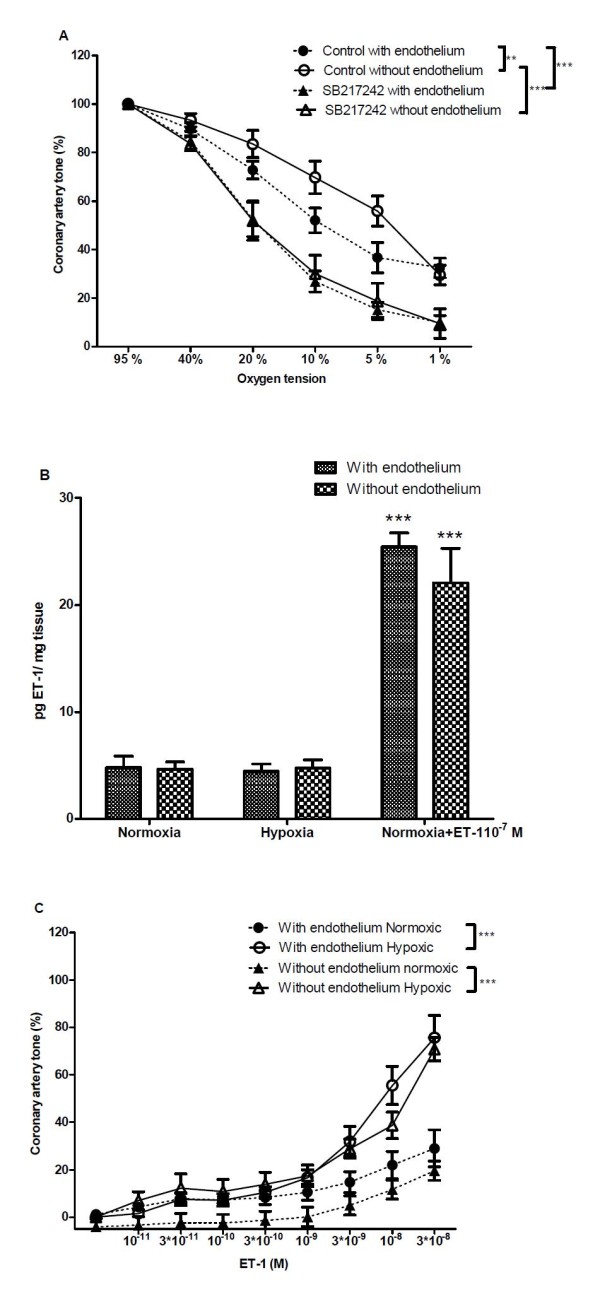
**Effect of the endothelin receptor antagonist SB217242 on hypoxic vasodilation**. (A) Effect of the endothelin receptor antagonist SB217242 (10 μM) on concentration-response curves for oxygen (O_2_) lowering in coronary arterial segments with and without endothelium contracted with PGF_2α _(10 μM). (B) Tissue ET-1 in arteries exposed to 1 h of hypoxia (5% CO_2_, 95% N_2_) compared to normoxic controls. By adding ET-1 (10^-7 ^M) to the organ bath we were able to measure ET-1 (P < 0.001). (C) Concentration-response curves for increasing concentrations of ET-1 in coronary artery segments with and without endothelium and constricted with PGF_2α _(10 μM) and subsequently exposed to 1% O_2 _or 95% O_2 _for 30 min as well as during the rest of the experiments. Results are means ± SEM of 4-9 experiments. Differences were evaluated with two-way ANOVA followed by Bonferroni post-test: * P < 0.05, ** P < 0.01, ***P < 0.001 compared to control.

In PGF_2α _(10 μM) contracted coronary arterial segments with endothelium lowering of O_2 _resulted in a transient increase in tension followed by relaxation which was most pronounced at 1% O_2 _(Figure 1A). In segments without endothelium, the concentration-response curve for lowering O_2 _was rightward shifted (Figure 1B, Figure 2A), but the relaxation induced by 1% O_2 _was similar in segments with (73.2 ± 7.2%, n = 5) and without endothelium (75.0 ± 3.1%, n = 4). In a pressure myograph, 1% O_2 _also increased diameter in segments with endothelium (19.4 ± 2.7%, n = 14) to a similar degree as in segments without endothelium (20.8 ± 2.6%, n = 14).

The endothelin receptor antagonist SB217242 significantly increased hypoxic relaxation regardless of the presence or absence of endothelium (Figure 2A). Addition of SB217242 to the organ bath resulted in less than 1% reduction in tone indicating limited ET-1 mediated basal vasoconstrictor tone in the coronary arteries. Tissue ET-1 was measured in arteries both with and without endothelium exposed to 1 hour of hypoxia compared to normoxic controls and no statistical differences in ET-1 concentration were found (Figure 2B). ET-1 added to the organ bath in control experiments could also be detected. Concentration response curves were constructed for ET-1, and we found ET-1 reversed vasodilation induced by 1% O_2 _in arteries with and without endothelium. The sensitivity towards ET-1 was increased at 1% O_2 _compared to 95% O_2 _both in arteries with and without endothelium (Figure 2C).

**Figure 3:**
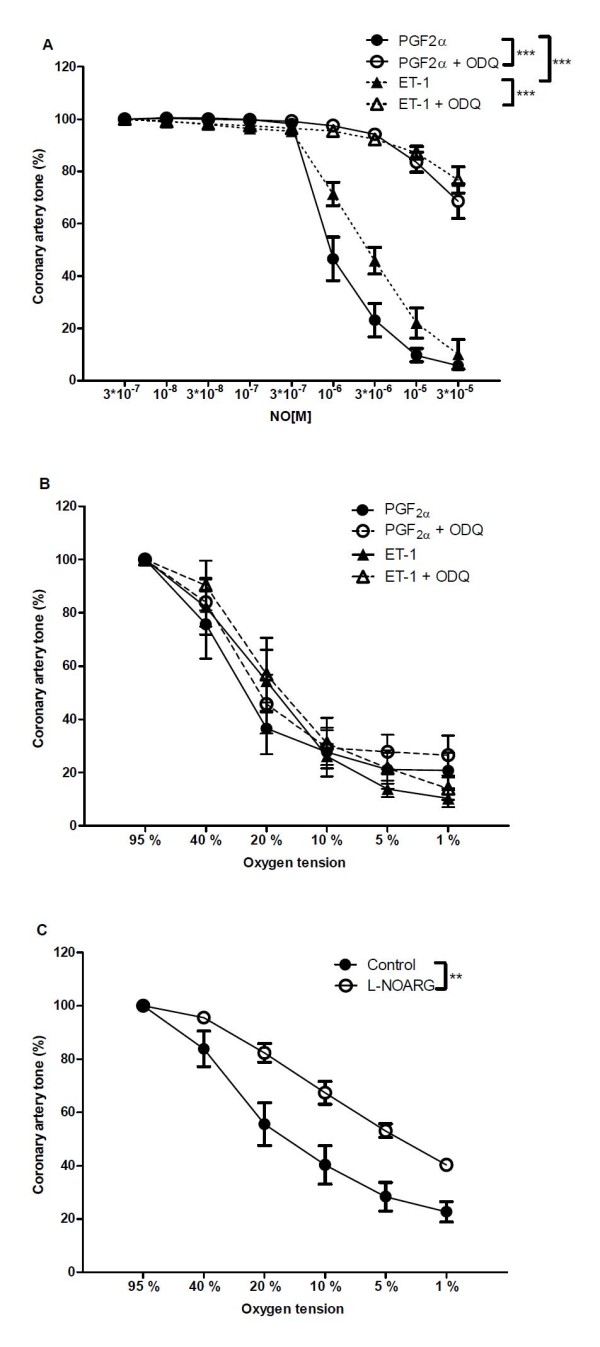
**Role of the nitric oxide (NO)-cyclic GMP pathway in hypoxic vasodilation**. (A) Concentration-response curves for NO in the absence and the presence of an inhibitor of soluble guanylyl cyclase, ODQ (3 × 10^-6 ^M) in arteries contracted with endothelin or PGF_2α_. (B) Concentration-response curves for O_2 _lowering in the absence and the presence of an inhibitor of soluble guanylyl cyclase, ODQ (3 × 10^-6 ^M) in arteries contracted with endothelin or PGF_2α _(C) Concentration-response curves for O_2 _lowering in the absence and the presence of an inhibitor of NO synthase, L-NOARG (3 × 10^-4 ^M). Results are means ± SEM of 6 experiments. Differences were evaluated with two-way ANOVA with Bonferroni post-test: * P < 0.05, ** P < 0.01, ***P < 0.001 compared to control.

Concentration-responses for exogenously added NO were rightward shifted in coronary arterial segments contracted to the same level with endothelin compared to PGF_2α _(Figure 3A). In PGF_2α _and ET-1 contracted arterial segments, an inhibitor of guanylyl cyclase, ODQ (3 μM) inhibited the response to exogenously added NO markedly (Figure 3A), but failed to change the response to O_2 _lowering (Figure 3B). Coronary arterial relaxation induced by O_2 _lowering from 95 to 1% O_2 _was reduced after inhibition of nitric oxide synthase with L-NOARG 300 μM (Figure 3C).

**Figure 4:**
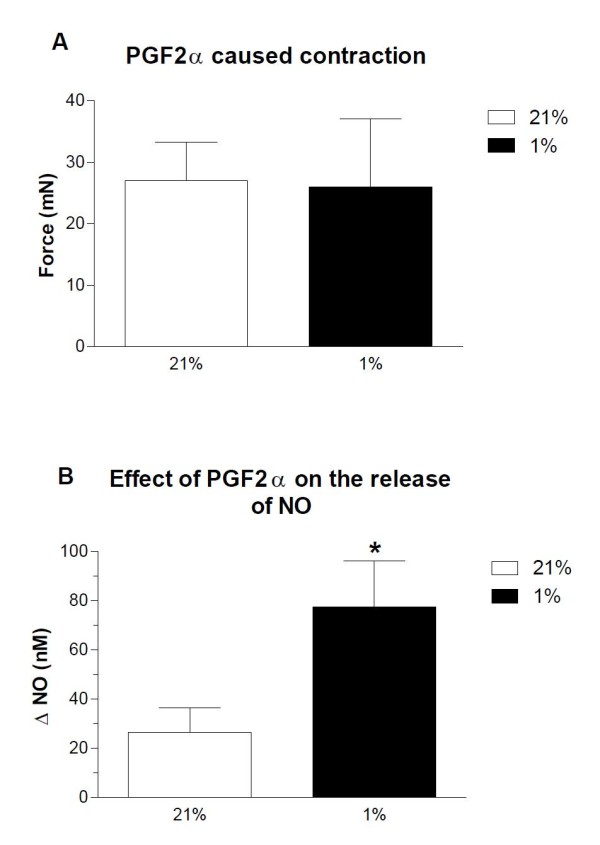
**Effect of acute hypoxia on nitric oxide release**. Effect of acute hypoxia (1% O_2_) on (A) constriction to PGF_2α _and (B) the release of NO. Results are means ± SEM of 5 experiments. Differences were evaluated with a paired t test: *P < 0.05 versus control,

Basal NO release was evaluated by the addition of a scavenger of NO, oxyhaemoglobin (10 μM) and basal release of NO was calculated as a reduction in electrode current as previously reported [19]. The procedure was performed during both normoxic and hypoxic conditions. There was no significant difference in basal release of NO at 21% O_2 _(64.2 ± 16 nM, n = 6) versus 1% O_2 _(64.3 ± 14 nM, n = 5). In arteries contracted to the same level by adding additional PGF_2α _(Figure 4A), the NO concentration was markedly enhanced at 1% O_2 _compared to 21% O_2 _(Figure 4B). Dialysate concentrations of the NO synthase inhibitor ADMA from arteries investigated in normal HEPES were at the lower detection limit (<0.06 μM) and showed no tendency to increase during hypoxia. We found that following the addition of ADMA 10 μM to the organ bath, significant amounts of ADMA could be recovered in the dialysate and this was independent of oxygenation (21% O_2_: 1.9 ± 0.3 μM; 1% O_2_: 2.0 ± 0.3 μM, n = 6).

**Figure 5:**
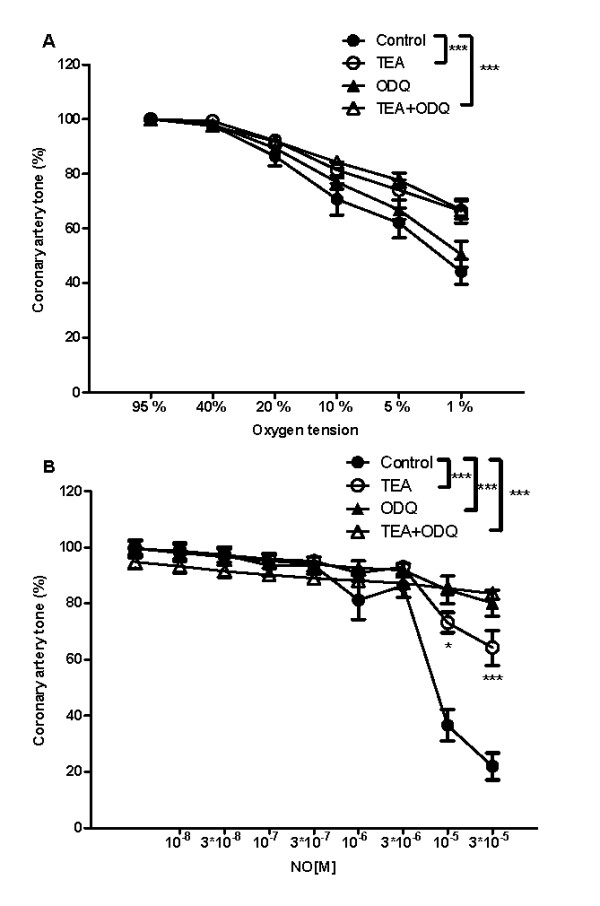
**Effect of the potassium channel blocker TEA on hypoxic vasodilation**. (A) Concentration-response curves for O_2 _lowering in the absence and the presence of an inhibitor of soluble guanylyl cyclase, ODQ (3 × 10^-6 ^M) and the absence or presence of the potassium channel blocker TEA (10 μM). (B) Concentration-response curves for NO in the absence and the presence of an inhibitor of soluble guanylyl cyclase, ODQ (3 × 10^-6 ^M) and the absence or presence of the potassium channel blocker TEA (10 μM). Results are means ± SEM of 8 experiments. Differences were evaluated with two-way ANOVA with Bonferroni post-test: * P < 0.05, ** P < 0.01, ***P < 0.001 compared to control.

A non-specific potassium channel blocker, TEA significantly inhibited relaxation induced by O_2 _lowering (Figure 5A). Concentration-responses for exogenously added NO were significantly inhibited in the presence of TEA (Figure 5B). A subanalysis showed that the presence of ODQ (3 μM) together with TEA gave an additional inhibition of NO induced relaxation as compared to TEA alone at 10^-5 ^and 3*10^-5 ^M NO.

**Figure 6:**
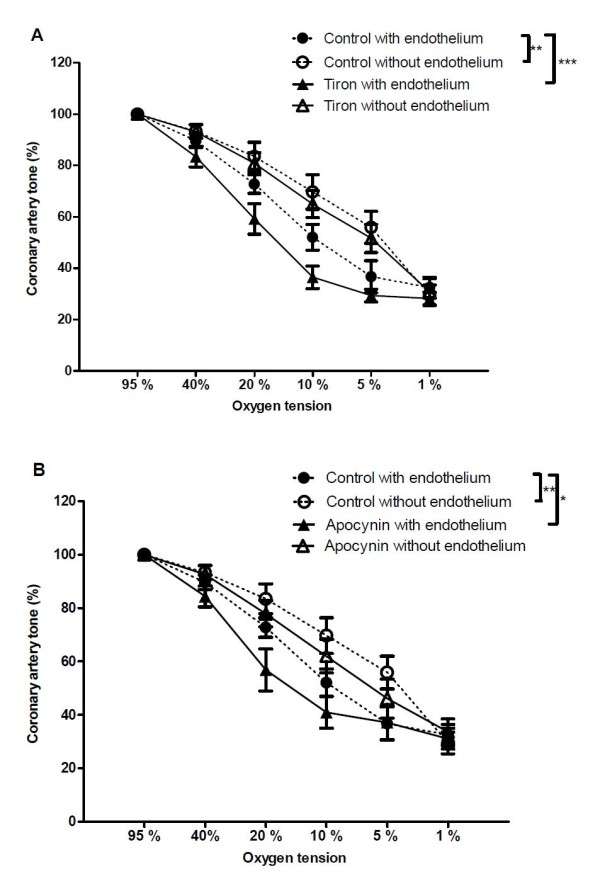
**Effect of the free radical scavenger tiron and the putative NADPH oxidase inhibitor apocynin on hypoxic vasodilation**. Effect of (A) the free radical scavenger tiron (10 μM) and (B) the putative NADPH oxidase inhibitor, apocynin (10 μM) on concentration-response curves for oxygen lowering in coronary arterial segments contracted with PGF_2α _(10 μM). Results are means ± SEM of 8-9 experiments. Differences were evaluated with two-way ANOVA with Bonferroni post-test: * P < 0.05, ** P < 0.01, ***P < 0.001 compared to control.

**Figure 7:**
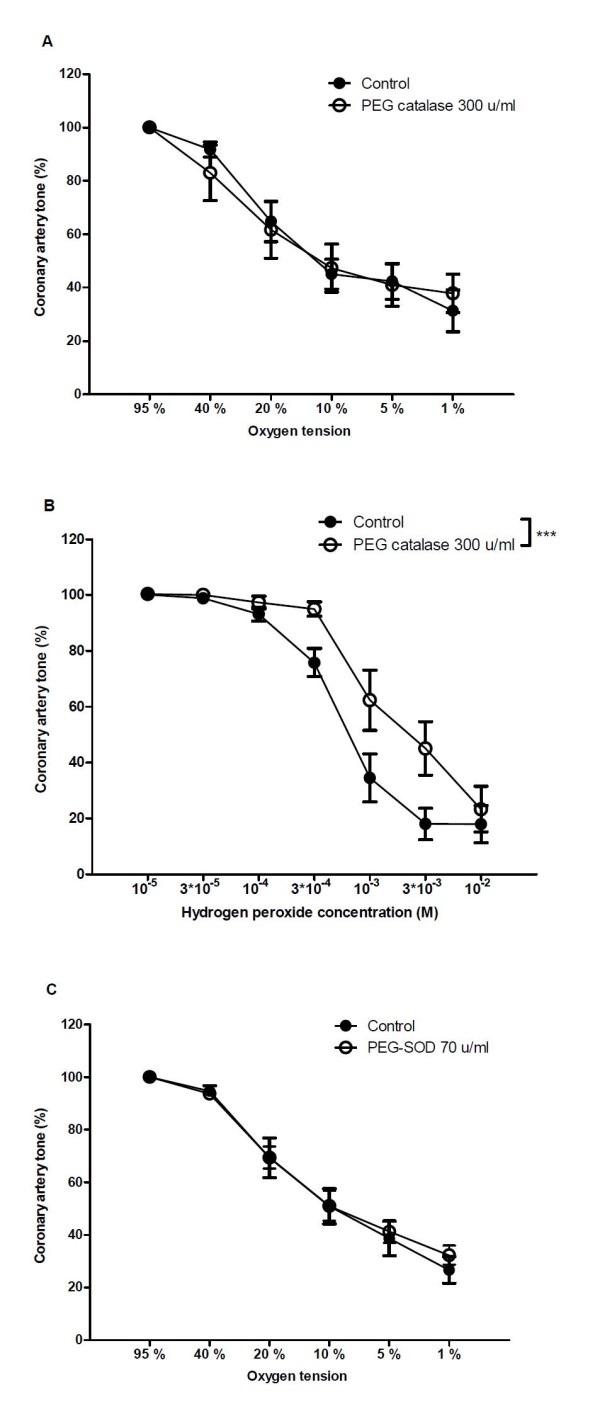
**Effect of PEG-catalase and PEG-SOD on hypoxic vasodilation**. (A) Effect of PEG-catalase (100 u/ml) on concentration-response curves for oxygen lowering and (B) Effect of PEG catalase (100 u/ml) on concentration-response curves for hydrogen peroxide. (C) Effect of PEG-SOD (70 u/ml) on concentration-response curves for oxygen lowering. Results are means ± SEM of 8 experiments. Differences were evaluated with two-way ANOVA followed by Bonferroni post-test: * P < 0.05, ** P < 0.01, ***P < 0.001 compared to control.

In arteries with endothelium, the transient contractions disappeared and a leftward shift in concentration-response curves for O_2 _lowering was observed in segments treated with the superoxide scavenger, tiron, while tiron had no effect in segments without endothelium (Figure 6A). In arterial segments with endothelium, an inhibitor of superoxide formation, apocynin leftward shifted concentration-response curves for O_2 _lowering, but this was not the case for arteries without endothelium (Figure 6B). The vasodilation to 1% O_2 _was unaltered in the presence of either tiron or apocynin. Additional tests of the potential role of H_2_O_2 _showed that the cell permeable enzyme PEG catalase 300 u/ml failed to change hypoxic relaxation (Figure 7A) while H_2_O_2 _-induced relaxation was inhibited (Figure 7B). A test for the potential role of superoxide showed that PEG-SOD 70 u/ml failed to change the curves for O_2 _lowering (Figure 7C).

**Figure 8:**
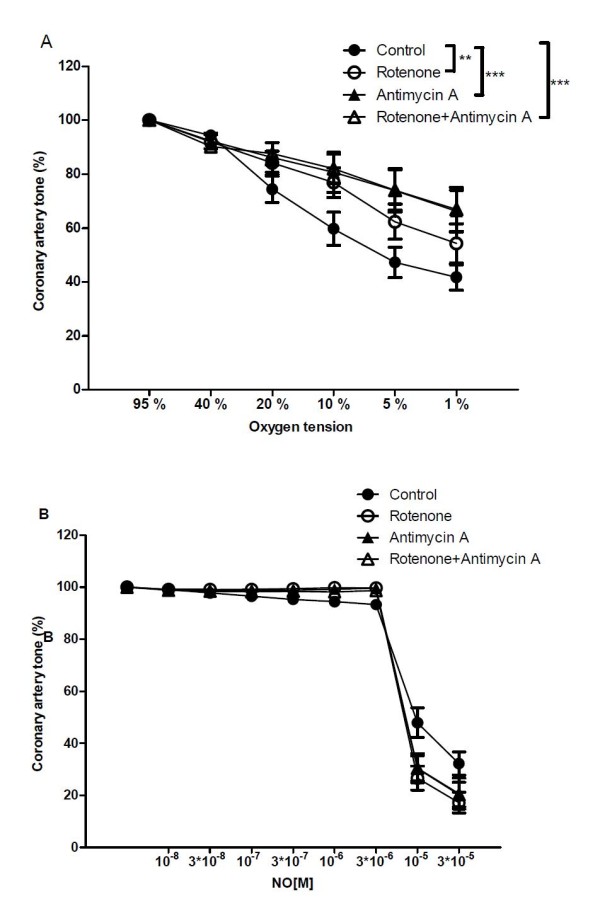
**Effect of Rotenone and Antimycin A on hypoxic vasodilation**. Effect of rotenone (1 μM), antimycin A (1 μM), and rotenone (1 μM)+ antimycin A (1 μM) on concentration-response curves for (A) oxygen lowering and (B) Concentration-response curves for NO. Results are means ± SEM of 9 experiments. Differences were evaluated with two-way ANOVA followed by Bonferroni post-test: * P < 0.05, ** P < 0.01, ***P < 0.001 compared to control.

The mitochondrial inhibitors rotenone (1 μM) and antimycin A (1 μM) both significantly inhibited vasodilatation to O_2 _lowering (Figure 8A). The combination of the two inhibitors did not have an additional effect compared to either of the inhibitors alone (Figure 8A). The mitochondrial inhibitors did not change NO relaxation (Figure 8B).

## Discussion

There are three major findings of the present in vitro investigation. First, endothelin receptor activation reverses while endothelin receptor antagonism markedly enhances hypoxic vasodilation in pig coronary arteries with and without endothelium. These findings suggest that endothelin counteracts hypoxic vasodilation in porcine coronary arteries independently of the endothelium. Second NO release was augmented during hypoxia and L-NOARG inhibited hypoxia-induced relaxation suggesting that NO contributes to hypoxic relaxation. Since a non-specific K channel blocker, TEA inhibited relaxations induced by O_2 _lowering or NO, the counteracting effect of endothelin on hypoxic vasodilation may take place through inhibition of K channels. Third, there was no additional release of ADMA or indication of additional release of oxygen free radicals during severe hypoxia (5 and 1% O_2_).

### Endothelin counteracts hypoxic vasodilation

Despite overall vasodilation to hypoxia, previous studies report increased endothelial release of ET-1 in response to hypoxia in systemic arteries [7,8], and hypoxia was suggested to increase ET-1 levels in cultured human umbilical venous endothelial cells exposed to 24 hours of hypoxia [26]. In addition to the endothelium, formation of endothelin can also take place in smooth muscle and adventitial cells of the vascular wall [27,28]. In the present study we were able to measure changes in endothelin concentration when it was added exogenously, but we did not observe any difference in the amount of ET-1 in porcine coronary arteries exposed to hypoxia. We cannot exclude the possibility that stoichiometric binding of endothelin may differ in normoxic versus hypoxic situations. Previous studies in rats show that most cardiac endothelin receptors are free of bound endothelin [29] and that ischemia-reperfusion increases ET-1 binding in rabbit hearts [30]. Such changes would not have been reflected in tissue endothelin concentrations as measured by our ELISA assay. However, the mixed endothelin receptor antagonist SB217242 (enrasentan) increased relaxation regardless of the presence or absence of endothelium. In previous studies we have found that endothelin contraction is antagonized by the ET_A _receptor antagonists BQ123 as well as by SB217242 [31] in the porcine coronary arteries [19].

Previous studies have suggested that exposure to hypoxic conditions may enhance the response to endothelin. Thus, in an in vitro study of pig coronary subendocardial arterioles two weeks after myocardial infarction an increased vasoconstrictor response to ET-1 was observed in vessels from animals with myocardial infarction compared to vessels from control animals [32], and an increased vasoconstriction to endothelin was found in the coronary arteries after ischemia-reperfusion of the porcine heart [33]. In the present study, addition of exogenous endothelin-1 reversed hypoxic vasodilation and endothelin contraction was enhanced in the arteries exposed to hypoxia. Together with enhanced vasorelaxation in the presence of an endothelin receptor antagonist, and the lack of change in vascular wall endothelin concentration, these findings suggest that endothelin counteracts hypoxic vasodilation probably due to an increased endothelin receptor activation of hypoxic arteries.

A non-endothelial origin of endothelin has previously been suggested based on the observation that the ET_A _antagonist BQ123 inhibited anoxic contractions in canine coronary arteries without endothelium [4]. In the present study, the non-endothelial origin of endothelin is supported by our observation that an endothelin receptor antagonist enhances relaxation to O_2 _lowering in arteries without endothelium and that contribution of endothelial-derived endothelin to the measured amount of endothelin in the porcine coronary arteries was insignificant (Figure 2).

### Role of NO in hypoxic vasodilatation

Studies using both wire-based and pressurised in vitro set-ups have found that inhibition of NO synthesis reduces hypoxic vasodilation [34-37], although L-NOARG had no effect on hypoxic relaxation in rat conduit coronary arteries [38]. Also, in vivo, Nase et al. [39] measured NO release in rat intestinal arterioles by means of microelectrodes and found a two-fold increase in arteriolar NO concentration during oxygen reduction. In the present study direct measurements of NO also suggest that NO increases in contracted arteries exposed to hypoxia. Together with the observation that the concentration-response curves for O_2 _lowering are rightward shifted by endothelial cell removal, and by an inhibitor of NO synthase, L-NOARG, these findings suggest that NO contributes to hypoxic vasodilation in porcine coronary arteries. However, experiments in arterial segments without endothelium or after inhibition of the NO-cGMP pathway in the present study, also revealed that smooth muscle vasodilatory pathways independent of the endothelial cell layer appear to contribute to hypoxic vasodilation in porcine large coronary arteries.

Following our recent findings that the plasma concentration of the endogenous NO synthase inhibitor, ADMA, rises in patients with myocardial infarction [13] and that ADMA reduces coronary artery contraction to *hyperoxia *[19] it was logical to investigate the role of ADMA in hypoxic coronary dilation. We were able to recover ADMA when it was added, but the concentration of ADMA was extremely low in coronary artery interstitial fluid and did not rise during hypoxia. Addition of pathophysiologically relevant concentrations (10^-5 ^M, derived from our previous human study [13]) of ADMA to the organ bath did not change the arterial response to hypoxia. Moreover, these concentrations of ADMA only cause incomplete inhibition of eNOS [21]. Therefore these findings suggest that ADMA in the blood stream does not appear to play a role in hypoxia-induced diameter changes.

It is well accepted that NO derived from the endothelium or drugs promotes vascular relaxation through stimulation of soluble guanylate cyclase and generation of cyclic GMP [40]. Hypoxia has also been found to increase cyclic GMP formation in bovine pulmonary arteries [41]. However, in contrast to inhibition of NO synthase with L-NOARG, inhibition of soluble guanylate cyclase by ODQ failed to reduce relaxations induced by O_2 _lowering, although NO relaxations were reduced. These findings suggest that endothelium-derived NO in hypoxic conditions may cause guanylate cyclase-independent relaxations.

The NO-cGMP pathway can lead to activation of smooth muscle ATP-sensitive and large-conductance calcium-activated K (BK_Ca_) channels both through protein kinase G dependent and independent pathways [42,43]. In the present study, the non-specific K channel blocker TEA inhibited relaxations induced by O_2 _lowering both in the absence and the presence of ODQ, and also inhibited relaxations induced by exogenously added NO. ODQ was reported to cause less inhibition of the NO donor, S-nitroso-N-acetylpencillamin-induced relaxation in bovine pulmonary arteries exposed to hypoxia [41], and together with our finding that activation of TEA-sensitive channels is involved both in the hypoxic and NO-induced relaxations in porcine coronary arteries, these findings may suggest that K channels are involved in the relaxations induced by the increased NO observed in acute hypoxia, although other mechanism such as NO-mediated inhibition of smooth muscle sarcoplasmic Ca-ATPase may also contribute [41]. Endothelin has also been shown to inhibit K_ATP _channel conductance [44], and BK channels [45] in porcine coronary artery smooth muscle cells. This latter effect of endothelin may explain how hypoxic relaxation is counteracted in porcine large coronary arteries.

### Role of ROS and mitochondrial enzymes in hypoxic vasodilation

ROS are natural by-products of the normal metabolism of oxygen and are essential in cell signalling. ROS include superoxide anion (O_2_^-^), the electronically excited singlet oxygen (^1^O_2_), hydrogen peroxide (H_2_O_2_), and hydroxyl radical (OH). Saitoh et al. [46] suggested that H_2_O_2 _is produced in a feed forward fashion in proportion to cardiac metabolism and is directly coupled to coronary blood flow [46]. Some studies support such a feed forward theory [47,48] while results from other studies oppose it [49,50]. In this study ROS inhibition with apocynin or tiron inhibited vasorelaxation to moderate O_2 _levels (10% O_2_), but failed to alter porcine coronary dilation in severe hypoxic conditions (1% O_2_). Moreover, the effect of tiron and apocynin was only present in segments with endothelium suggesting that in moderate hypoxic conditions ROS formation may counteract hypoxic vasodilation by reacting with endothelium-derived NO. Superoxide dismutase and catalase scavenge, respectively, extracellular O_2_^- ^and H_2_O_2 _and in the pegylated forms, the penetration into the vascular wall is increased of these molecules. However, PEG-SOD and PEG-catalase failed to change relaxations induced by O_2 _lowering, although PEG-catalase inhibited exogenously added H_2_O_2_. These results agree with recent findings in fetal chicken femoral arteries where PEG-SOD and PEG-catalase also failed to change relaxation in severe hypoxia [51]. Although we cannot exclude non-specific effects of tiron and apocynin [52,53], the effects of both tiron and apocynin appear to be specific, since the effects of moderate O_2 _levels are only observed in segments with endothelium. Therefore, a likely explanation for these results is that the reaction of NO and O_2_^- ^to form ONOO^- ^is too fast to allow the reaction of O_2_^- ^with the antioxidant e.g. PEG-SOD in the vascular wall [54]. However, overall the results of the present study suggest that ROS do not appear to play a major role in relaxations associated with severe hypoxia.

The mitochondrial electron transport chain is in hypoxic conditions both considered as O_2 _sensor and a source of NO accumulation due to decreased metabolization of NO by cytochrome C in a reduced state [55,56]. Hypoxia-induced relaxations have previously been reported to be reduced by inhibitors of the mitochondrial electron transport chain complex I (rotenone), complex III (myxothiazole and antimycin A), and complex IV (NaN_3_) [51,57]. In the present study both rotenone and antimycin A reduced relaxation induced by O_2 _lowering. Rotenone and antimycin A were also found to increase formation of O_2_^- ^in bovine coronary arteries [57]. However, in the present study rotenone and antimycin A did not change relaxations induced by exogenously added NO, and hence NO bioavailability. Therefore, our results do not suggest a role for ROS in severe hypoxia in porcine coronary arteries. Thus, our results agree with observations in bovine coronary arteries that ROS are not primary mediators of the hypoxic relaxation, but sustain the presence of an O_2 _sensing/signaling model based on mitochondrial control of pyruvate metabolism associated with cytosolic NADPH redox regulation [55,57]. In addition to the mitochondrial O_2 _sensing/signaling in smooth muscle relaxation, our findings of increased NO formation in hypoxic conditions (Figure 4), support an endothelial mitochondrial sensing mechanism leading to increased NO formation.

## Conclusions

The present results in porcine coronary arteries suggest NO contributes to hypoxic vasodilation, probably through the K channels, which is reversed by addition of ET-1 and enhanced by endothelin receptor antagonism. These latter findings suggest that endothelin receptor activation counteracts hypoxic vasodilation.

The dilatory response to hypoxia is important in the resistance arteries of the coronary circulation, but the dilatory response to hypoxia is also present in conduit vessels [1,58,59]. Combined with the fact that coronary atherosclerosis is confined to epicardial arteries, even minor diameter changes become highly important when a hemodynamically significant stenosis is present. In quantitative terms other scientific groups as well as ourselves typically find a 20% increase in conductance coronary artery diameter during hypoxia (the pressure myograph findings in this study). According to Poiseuille's law this equals a doubling in flow rate. In heart disease hypoxia is also a prominent feature of heart failure, but while local hypoxia may alter cellular function in the myocardium, systemic hypoxia may improve ventricular function secondary to changes in vascular tone [60]. In the atherosclerotic human coronary circulation endothelium-dependent vasodilation is impaired [61] and this is associated with an increased frequency of cardiac events. Release of endothelium-derived NO is decreased in endothelial dysfunction and could thus not contribute to hypoxic vasodilation and counteract the contractile effect of ET-1. Based on the present preclinical findings it would be interesting to investigate the therapeutic potential of endothelin receptor antagonists in ischemic heart disease although unexpected hemodynamic changes have been reported previously [62].

## Authors' contributions

ERH carried out the ET-1 measurements and myography studies, and performed statistical analysis and participated in study design and writing of the manuscript. ES carried out the NO measurements and performed some of the statistical analysis. US and OF participated in the design and idea of the study as well as in the writing of the manuscript. All authors have read and approved the final manuscript.
